# Underestimation about the Contribution of Nitrate Reducers to Iron Cycling Indicated by *Enterobacter* Strain

**DOI:** 10.3390/molecules27175581

**Published:** 2022-08-30

**Authors:** Ming-Jun Li, Meng-Yun Wei, Xiao-Ting Fan, Guo-Wei Zhou

**Affiliations:** 1 School of Resources and Environmental Engineering, Anhui University, Hefei 230601, China; 2 Key Lab of Urban Environment and Health, Institute of Urban Environment, Chinese Academy of Sciences, Xiamen 361021, China

**Keywords:** nitrate reducers, *Enterobacter*, enzymatic NRFO, nitrate reductases, iron cycling

## Abstract

Nitrate-reducing iron(II) oxidation (NRFO) has been intensively reported in various bacteria. Iron(II) oxidation is found to be involved in both enzymatic and chemical reactions in nitrate-reducing Fe(II)-oxidizing microorganisms (NRFOMs). However, little is known about the relative contribution of biotic and abiotic reactions to iron(II) oxidation for the common nitrate reducers during the NRFO process. In this study, the typical nitrate reducers, four *Enterobacter* strains *E. hormaechei*, *E. tabaci*, *E. mori* and *E. asburiae*, were utilized as the model microorganisms. The comparison of the kinetics of nitrate, iron(II) and nitrite and N_2_O production in setups with and without iron(II) indicates a mixture of enzymatic and abiotic oxidation of iron(II) in all four *Enterobacter* strains. It was estimated that 22−29% of total oxidized iron(II) was coupled to microbial nitrate reduction by *E. hormaechei*, *E. tabaci*, *E. mori*, and *E. asburiae*. *Enterobacter* strains displayed an metabolic inactivity with heavy iron(III) encrustation on the cell surface in the NRFOmedium during days of incubation. Moreover, both respiratory and periplasmic nitrate-reducing genes are encoded by genomes of *Enterobacter* strains, suggesting that cell encrustation may occur with periplasmic iron(III) oxide precipitation as well as the surface iron(II) mineral coating for nitrate reducers. Overall, this study clarified the potential role of nitrate reducers in the biochemical cycling of iron under anoxic conditions, in turn, re-shaping their activity during denitrification because of cell encrustation with iron(III) minerals.

## 1. Introduction

Nitrate-reducing Fe(II) oxidation (NRFO) with the production of iron(III), NO_2_^−^, NO, N_2_O and N_2_ have been found in soil, fresh water and brackish water [1]. NRFO is of great importance in driving iron biogeochemical cycling and removing nitrogen, metal(loid)s and radionuclides from the active sledge system and groundwater, respectively [2,3].

There are a lot of bacteria isolated with the activity of NRFO, such as *Acidovorax*, *Microbacterium*, *Pseudomonas*, *Citrobacter*, *Enterobacteriaceae* spp., etc. [4,5,6,7]. Nevertheless, the mechanism around the Fe(II) oxidoreductase involved in nitrate reduction coupled to iron(II) oxidation is still needed to be deciphered. Moreover, chemodenitrification (4Fe(II) + 2NO_2_^−^ + 5H_2_O → 10FeO(OH) + N_2_O + 6H^+^) complicates our understanding of the NRFO process, which always leads to a combination of biotic with abiotic iron(II) oxidation during NRFO process [2]. Up to date, all isolated neutrophilic nitrate-reducing Fe(II)-oxidizing microorganisms (NRFOMs) are mixotrophic, requiring an organic co-substrate for their continuous cultivation and oxidation of Fe(II) to Fe(III) [2,4,5,6]. It provides a cue that respiratory nitrate reduction is essential for the capability of NRFO by these NRFOMs. As a result, nitrite is an unavoidable by-product followed by a chemical reaction with iron(II) in the system, which is a challenge to the existence of enzymatic NRFO. Moreover, NRFO displays an intrinsic capability in all nitrate reducers, which directly implicates catalysis by nitrogen oxide reductases in cells and does not require a specialized oxidoreductase. Ishii et al. have indicated non-enzymatic iron(II) oxidation from 67 members affiliated with *Pseudogulbenkiania*, which are characterized as denitrifier [8,9]. However, this conclusion is arbitrary since there is no comparison of nitrate-reducing extent between setups amended with or without iron(II). Inconsistently, Jamieson et al. estimated that 60–70% of overall iron(II) oxidation is caused by an enzymatic pathway for NRFOMs including *Acidovorax* strain BoFeN1 and 2AN, *A. ebreus* strain TPSY, *Paracoccus denitrificans* Pd 1222 and *Pesudogulbenkiania* sp. strain 2002, which is depended on compilation and model-based interpretation from published experimental data [2]. It has been estimated that only 30–40% of oxidized iron(III) is owing to the chemodenitrification process for these strains [2]. However, it is still unknown about this discrepancy between NRFOMs and common nitrate reducers. Therefore, more research is still necessary to unveil the relative contribution of enzymatic reaction and chemodenitrification to iron(III) oxidation, especially for the common nitrate reducers.

Cell encrustation is observed on the surface or in the periplasmic space of NRFOMs and nitrate reducers, consequently, causing physical inactivity including termination of nutrition uptake and further cell metabolism [1,10,11,12]. This is likely to result in the re-estimation of their contribution to iron and nitrogen cycling. The position of cell encrustation actually reflects the types of nitrate reductases expressed by these microorganisms, including the respiratory nitrate reductases and periplasmic nitrate reductases [13]. According to the previous reports, substantial numbers and high diversity of nitrate reducers are widely distributed in various environments under anoxic or aerobic conditions [14], whereas, the atlas for the composition of nitrate reductases for these common nitrate reducers needs to be established.

The genus *Enterobacter*, which belongs to the family Enterbacteriaceae, is abundant in various environments such as soil, water and gut of soil animals [15]. *Enterobacter* spp. are typical nitrate reducers and can be used as model nitrate reducers [16]. In this study, four *Enterobacter* strains were employed, aiming to explore the style of their roles in iron(II) oxidation during the NRFO process. Moreover, the published documentation and public database were referred to construct a heatmap of nitrate-reducing genes possessed in Enterbacteriaceae bacteria, further investigating the potential position of cell encrustation formed for the bacterial cells after NRFO.

## 2. Results

### 2.1. Nitrate Reduction and Iron(II) Oxidation by Enterobacter Strains

**Figure 1:**
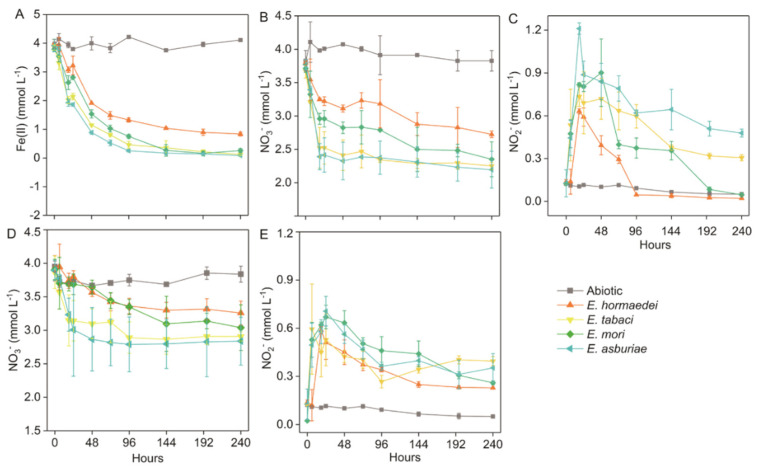
Time course of Fe(II), NO_3_^−^ and NO_2_^−^ for *Enterobacter* strains in the NRFO medium containing with (**A**−**C**) and without (**D**,**E**) iron(II) during the incubation. The error bars indicated the standard deviations of three replications.

There were four *Enterobacter* strains, including *hormaechei*, *E. tabaci*, and *E. asburiae*, used in this study (Table 1 and Supplementary Figure S1). Compared to abiotic setups, Fe(II) and NO_3_^−^ concentrations decreased with incubation for all groups inoculated with *Enterobacter* strains in the NRFO medium. The concentration of Fe(II) decreased sharply within 48 h and was then kept stable after 96-h incubation for all strains in the NRFO medium during the incubation (Figure 1A). Similarly, the time course of nitrate concentrations shared a similar trend with that of Fe(II) concentration for all biotic setups in the NRFO medium during the incubation (Figure 1B). For the nitrite concentrations, all biotic setups were observed to be rapidly elevated in the first 18 h and then decreased until exhaustion in addition to the amendment inoculated with *E. tabaci* and in the NRFO medium during the incubation (Figure 1C). For the NRFO medium without iron(II) addition, the decrease in nitrate and nitrite was also identified for all biotic setups amended with *Enterobacter* strains during the incubation, however, which displayed a lower extent than those in setups amended with iron(II) (Figure 1D,E). There was a hundred micromolar of nitrite remaining in the medium after 240-h incubation (Figure 1E).

**Figure 2:**
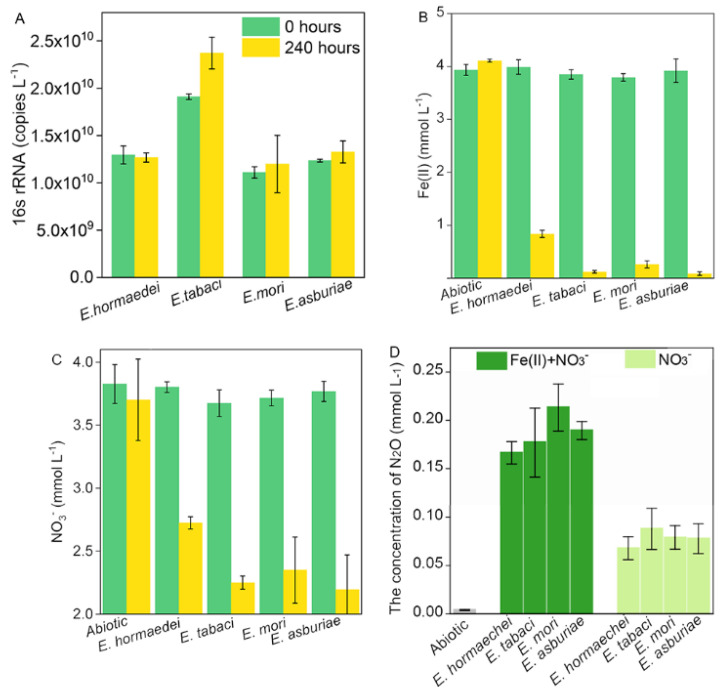
The ability of iron(II) oxidization and nitrate reduction by *Enterobacter* strains in the NRFO medium after 240 h of incubation. (**A**) The number of cells for *Enterobacter* strains in the NRFO medium. (**B**) The extent of iron(II) oxidation by *Enterobacter* strains in the NRFO medium during 10−day incubation. (**C**) The extent of nitrate reduction by *Enterobacter* strains in the NRFO medium during 10−day incubation. (**D**) The concentration of N_2_O in the NRFO medium after 10−day incubation. “Fe(II)+NO_3_^−^” and “NO_3_^−^” indicated the medium containing with and without iron(II) during the incubation.

The cell copy numbers of *Enterobacter* strains ranged from 8.68 × 10^9^ to 1.81 × 10^10^ at the beginning of the incubation, and they slightly increased after 240 h in addition to setups amended with *E*. *hormaechei* (Figure 2A). In comparison, strain *E. tabaci* and *E. asburiae* displayed higher extents of iron(II) oxidation and nitrate reduction than *E. hormaechei* and *E. mori* (Figure 2B). The strain *E. tabaci*, *E. mori* and *E. asburiae* possessed the ability to almost completely oxidize iron(II), while there was around 0.84 mM iron(II) remaining in the medium for *E. hormaechei* after 240-h incubation (Figure 2B). Moreover, all the strains showed incomplete nitrate reduction after 240 h (Figure 2C). Additionally, the nitrite content of *E. hormaechei* and *E. mori* approached 0 mM, while 0.31 mM and 0.48 mM of nitrite were still contained in the medium for biotic setups inoculated with strain *E. tabaci* and *E. asburiae* after 240 h, respectively (Figure 1C). The production of N_2_O was detected in all biotic treatments containing with and without iron(II) (Figure 2D). In comparison, the concentration of headspace N_2_O was higher in the iron(II)-containing setups than that in biotic treatments without iron(II) (Figure 2D).

### 2.2. Morphological Characteristics of Enterobacter Strains after Iron(II) Oxidation and Nitrate Reduction

**Figure 3:**
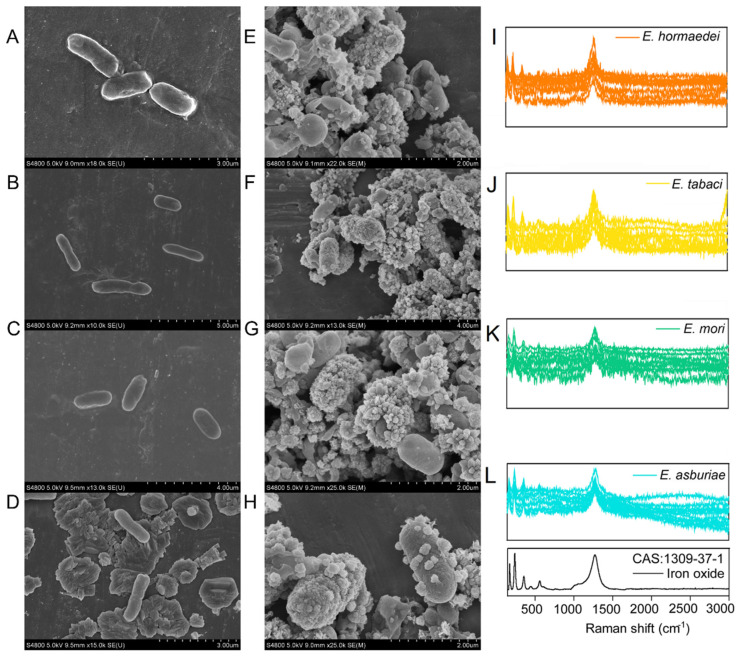
Morphological characteristics of *Enterobacter* strains in the LB (**A**–**D**) and NRFO (**E**–**H**) medium and iron(III) oxides (**I**–**L**) in the surface of the corresponding cells using Raman microspectroscopy.

It was observed that cells of these four strains were smooth on the surface when they were cultivated in the R2A medium (Figure 3A–D). Whereas, cells of them were identified to be covered by heavy encrustation after incubation in the NRFO medium (Figure 3E–H). In order to further characterize the coat on the surface of cells, Raman spectroscopy was employed. Spectra of all cells from these four strains were consistent with that of the standard iron oxide (Figure 3C).

In order to investigate the metabolic activity of *Enterobacter* strains, C-D bond (2040 and 2300 cm^−1^) was detected for cells of *Enterobacter* strains after incubation in the NRFO medium. For the cells coated with encrustation, there were no detectable peaks in the region between 2040 and 2300 cm^−1^ (Supplementary Figure S2).

### 2.3. Nitrate Reductase Contained in the Enterobacteriaceae Strains

**Figure 4:**
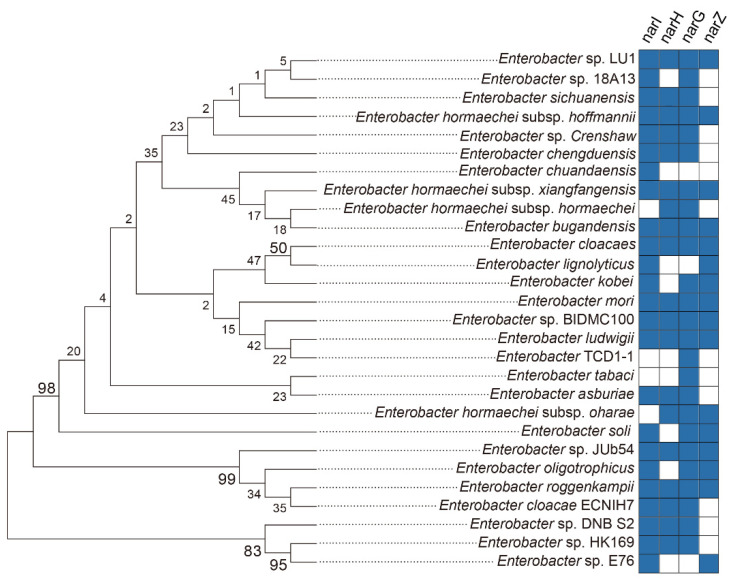
Nitrate-reducing genes harbored in the *Enterobacter* strains according to the NCBI database. Neighbor-joining (bootstrap: 1000 replicates) phylogenetic tree based on 16S *rRNA* gene sequences indicating the phylogenetic position of the *Enterobacter* strains. Bar represents 1 substitution per 100 nucleotide positions. Bootstrap values above 50% are given at the node.

Nitrate reduction-relevant genes, including *narG*, *nasA*, *nirK*, *nirS*, *norB* and *nosZ*, were detected in genomes of these four *Enterobacter* strains (Table 2). In order to investigate the potential capability of nitrate reduction for the genus *Enterobacter*, four nitrate reductase-encoding genes, including *narI*, *narH*, *n*,*arG* and *narZ*, were investigated in the genomes of 28 *Enterobacter* strains (Figure 4 and Supplementary Table S2). For the gene *narI*, 24 strains were found to include this gene in their genomes in addition to *E. Hormaechei* subsp. *hormaechei*, *E. Hormaechei* subsp. *oharae* and *E. tabaci* (Figure 4 and Supplementary Table S2). In comparison, there were 19, 25, and 16 strains encoding *narH*, *narG* and *narZ* genes in their genomes, respectively (Figure 4 and Supplementary Table S2).

To further explore the possibility of nitrate reduction by Enterobacteriaceae bacteria in environments, three types of nitrate reductases, including respiratory nitrate reductase 1, 2 and periplasmic nitrate reductase, were searched depended on the NCBI database. For the respiratory nitrate reductase 1, consisting of four subunits such as NarG, NarH, NarI and NarJ, it was more abundant in Enterobacteriaceae spp. than the respiratory nitrate reductase 2 and periplasmic nitrate reductase (Supplementary Figure S3 and Supplementary Table S3). Three subunits (e.g., NarV, N,arY and NarZ) make up the respiratory nitrate reductase 2, which were widely identified in genomes of Enterobacteriaceae bacteria (Supplementary Figure S3 and Supplementary Table S3). In comparison, around 7 genera, e.g., *Shigella*, *Escherichia*, *Salmonella*, *Citrobacter*, etc., possess genes encoding the subunits of periplasmic nitrate reductase containing NapA, NapB, NapC, NapD and NapF (Supplementary Figure S3 and Supplementary Table S3).

## 3. Discussion

### 3.1. Microbial-Mediated Nitrate-Dependent Fe(II) Oxidation by Enterobacter Strains

Aiming to unveil the ability of NRFO by *Enterobacter* spp., four strains were chosen to perform the incubation. No organic electron donor was added in the NRFO medium. In comparison with setups amended with both Fe(II) and nitrate, reduced nitrate and nitrite were also detected in biotic setups without the addition of iron(II) (Figure 1D,E). However, contents of consumed nitrate and nitrite in iron(II)-absent treatments were markedly lower than those in setups amended with Fe(II) (Figure 1D,E). For one thing, it meant that these strains probably employed the stored endogenous organic carbon within cells, which may be generated during the pre-cultivation of *Enterobacter* strains in the R2A medium. It has been documented that endogenous carbon could perform an as energy source for denitrification when the exogenous organic substrates are exhausted [17,18,19,20,21,22,23,24,25,26,27,28,29,30,31,32,33,34,35,36,37,38,39,40,41,42]. Also, the remaining NO_2_^−^ (0.23−0.39 mM) in the incubation (Figure 1E) indicated that limited endogenous organic carbon was not sufficient enough to support the complete reduction of nitrite. For the other, part of reduced nitrate was considered to be coupled to microbial iron(II) oxidation by *Enterobacter* strains in setups amended with both nitrate and iron(II) because the abiotic reaction between iron(II) and nitrate is slow [9]. The chemical equation is as follows [43].

NO_3_^−^ + 2Fe^2+^ + 2H^+^ → 2Fe^3+^ + NO_2_^−^ + H_2_O (1)

2NO_2_^−^ + 4Fe^2+^ + 6H^+^ → N_2_O + 4 Fe^3+^ + 3H_2_O (2)

During the incubation, a total of 1.08−1.61 mM nitrate was consumed in setups amended with iron(II), whereas the amount of reduced nitrate was 0.67−1.06 mM in iron(II)-absent setups (Figure 1). It was roughly estimated that a small part of nitrate (0.41−0.55 mM) reduction (34–38%) was microbially driven by the exogenous donor-iron(II). In the term of enzymatic NRFO, nitrate is assumed to be exclusively reduced to nitrite [2]. Based on the ratio of nitrate to iron(II) [stoichiometric equations of NRFO (Equation (1))], 0.82−1.10 mM iron(II) was thought to be coupled to biotic nitrate reduction, which accounted for 22−29% of total oxidized iron(II) during the incubation for the strains *E. hormaechei*, *E. tabaci*, *E. mori* and *E. asburiae*, respectively (Figure 1).

Nitrite concentration fluctuated in all biotic setups without iron(II) during the incubation was likely to be attributed to the microbial reduction of nitrite by *Enterobacter* strains, which was agreed with the relevant function genes-*nirK* and *nirS* were found from their genomes (Table 2). However, the extent of nitrite reduction was stopped after around 96 h of incubation (Figure 1F). It may be due to limited endogenous organic electron donors reserved with the in cells of microorganisms. In contrast, more nitrite was utilized during the incubation added with iron(II) (Figure 1C), which indicated that decrease of nitrite could result from the mixture of microbial catalyzation and chemodenitrification during the incubation. The amount of N_2_O, which was higher in the setups with iron(II) than in the iron(II)-absent ones (Figure 2D), was consistent with the consumed nitrite in biotic setups with and without iron(II). It further suggested a mixed microbial nitrite reduction and chemodenitrification (Equation (2)) during the NRFO incubation. Correspondingly, 2.70−2.98 mM Fe(II) would be chemically oxidized by NO_2_^−^ in the setups amended with Fe(II) and nitrate, contributing 71−78% of total iron(II) depleted during the incubation. This result presented a different relative contribution of biotic iron(II) oxidation and chemdenitrification to the iron(II) oxidation from the incubation with several mixotrophic NRFO bacteria including *Acidovorax* strain BoFeN1, 2AN, *A. ebreus* strain TPSY, *Paracoccus denitrificans* Pd 1222, and *Pseudogulbenkiania* sp. strain 2002. They have been demonstrated that about 60–75% of overall iron(II) oxidation was owing to the biotic process, and the section of organic ligands and exopolymeric substances by these bacteria can enhance abiotic oxidation of iron(II).

### 3.2. Cell Encrustation and Physical Inactivity after NRFO Process by Enterobacter Strains

It can be found that more than 2 mM nitrate was still kept in the medium for all the biotic setups after 240 h of incubation, indicating that an insufficient electron donor was included in the medium. However, there was around 0.12−0.88 molar of iron(II) remaining in the incubation for all the setups with four *Enterobacter* strains. Moreover, nitrate reduction almost ceased after 144 h for the biotic setups with *E. hormaechei*, *E. tabaci* and *E. asburiae* and 192 h for the biotic setups with *E. mori* (Figure 1B), which was almost at the same time with iron(II) oxidation (Figure 1A,B). In addition, the kinetic of nitrate exhibited the same trend as that of iron(II) during the incubation (Figure 1). Moreover, the was no large increase in the cell numbers of *Enterobacter* strains during 240-h incubation (Supplementary Table S2). All these *Enterobacter* strains seemed to enter a state of physical inactivity after hundred hours of NRFO reaction. Heavy cell encrustation by iron(III) oxides (Figure 3) can explain the loss of metabolic activity of *Enterobacter* strains. Furthermore, in order to verify the physical dormancy of *Enterobacter*, (H^2^)D_2_O-based Raman microscopy had been employed and no C-D bonds (ranging from 2040 to 2300 cm^−1^) were detected in all cells of iron(II)-amended setups after 240-h incubation (Supplementary Figure S2). This phenomenon agreed with previous reports about *Bacillus ferroxidans*, *Dechloromonas* sp. UWNR4, *Acidovorax* sp. 2AN [3,4,44]. It can speculate that the heavy encrustation on the surface of cells was the culprit for preventing the utilization of water and other substrates including iron(II), nitrate, and nitrite for cells of *Enterobacter* strains. The potential product NO generated from chemodenitrification is also toxic and can react with metalloproteins in the components of the electron transport chain [9,45], which may also contribute to the inactivity of *Enterobacter* strains. The cell encrustation of iron(III) oxides is identified as the artifacts of an abiotic reaction between sorbed Fe^2+^ and biogenic NO_2_^−^ in batch experiments [44]. The increase in the Fe(II) concentration (higher than 800 mg/L) can enhance the extent of cell encrustation in a continuous up-flow biofilter, whereas Fe(II)EDTA would prevent cells from encrustation and the nitrogen removal efficiency is as high as 90% [44]. Although numerous literatures indicated that microbial NRFO may result in cell crustation [46,47,48,49], *Paracoccus versutus* LYM is found with no cell encrustation formed when amended with organic co-substrate and Fe(II)EDTA was amended as electron donor [50]. *Enterobacter* strains in this study were encrusted by the mixed abiotic and biotic NRFO processes under the condition of no addition of exogenous organic matter. Our previous results demonstrated that encrusted and metabolic inactive cells of mixotrophic NRFO bacteria-*Bacillus ferroxidans* can re-awake and take off the iron(III) encrustation after re-incubation of these cells in the organics-abundant medium without the addition of iron(II) [51]. These suggested that the metabolic state of denitrifers or nitrate reducers might shift between inactive cells and active ones without encrustation inhibition in natural environments, such as the flooding and drainage of paddy soils.

### 3.3. The Potential Contribution of Nitrate Reducers to Iron Cycling Implicated by Enterobacter Strains

In this study, *Enterobacter* strains mediated NRFO is indicated to result in a combination of microbial iron(II) oxidation coupled to nitrate reduction with chemodenitrification by nitrite and iron(II), together contributing to iron(II) consumption during the incubation. It provides an insight into the potential of nitrate reducers or denitrifiers to markedly drive iron cycling via chemodenitrification, which is triggered by nitrite catalyzed by nitrate reductase Nar in iron(II)-rich environments such as flooded paddy soils. Most of *Enterobacteriaceae* strains were found to possess both respiratory nitrate reductase 1 or 2 (Supplementary Figure S3), locating the intracellular membrane of microorganisms. Respiratory nitrate reductases 1 and 2 are responsible for nitrate reduction activity when cells are grown anaerobically and aerobically in nitrate-containing environments, respectively [52]. It suggested that most of the *Enterobacter* strains could mediate nitrate reduction whether under anaerobic or aerobic conditions. The iron(III) oxides encrusted on cell surfaces of *Enterobacter* strains in this study suggested that microbial NRFO might depend on the a dedicated Fe(II) oxidoreductase according to Clark’s study, which would be followed by proton motive force generation and ATP production [9]. Although cells of *Enterobacter* strain entered into a state of metabolic inactivity, the NRFO process could be an alternative strategy to obtain energy for the nitrate reducers in the short term under an environment contained limited organic substrates [42]. In comparison, intracellular reactions between Fe(II) and periplasmic nitrate reductase (Nap) are essential to initiate extensive NRFO, which consumes periplasmic protons to reduce nitrate without energetic benefit [9,53]. Several genera harboring genes (*napA*, *napB*, *napC*, *napD* and *napF*) encoding periplasmic nitrate reductase (Supplementary Figure S3 and Supplementary Table S3), similarly, it could pose periplasmic iron(III) precipitation and limitation of carbon corporation [53]. Hence, these highly diverse and abundant nitrate reducers ubiquitously distributed in soils and aquatic environments, may contribute extremely to iron cycling.

## 4. Materials and Methods

### 4.1. Isolation of Nitrate-Dependent Fe(II)-Oxidizing Bacterium

Four strains are widely distributed in diverse environments such as soils and waters [17,18,19,20], including *Enterobacter hormaechei* (CGMCC 1.10608^T^), *E.* tabaci (CGMCC 1.15707^T^), *E.* mori (CGMCC 1.10322^T^) and *E.* asburiae (JCM 6501^T^) were purchased from Japan Collection of Microorganisms (JCM) and China General Microbiological Culture Collection Center (CGMCC). R2A medium [21] was used for routine cultivation under the oxic condition of these four strains affiliated with *Enterobacter*.

### 4.2. Experimental Setup

Nitrate-reducing Fe(II)-oxidization (NRFO) medium was employed for investigating the ability to oxidize Fe(II) by *Enterobacter* strains in this study. NRFO medium was constituted of mineral medium (pH 6.8–7.2), 10 mM NaNO_3_, 10 mM FeCl_2_, vitamin solution (1 mL L^−1^) [22], trace element solution SL10 (1 mL L^−1^) [22], selenite-tungstate solution (1 mL L^−1^) [10] and bicarbonate buffer (22 mM), which were prepared according to previous description [4,11]. The mineral medium included MgSO_4_·7H_2_O (0.5 g L^−1^), CaCl_2_·2H_2_O (0.1 g L^−1^), NH_4_Cl (0.3 g L^−1^) and KH_2_PO_4_ (0.6 g L^−1^) [23,24,25]. FeCl_2_ was added to the mineral medium in the anaerobic chamber after the mineral medium autoclaved (120 °C for 20 min) and cooled to room temperature under N_2_/CO_2_ (80/20%) [4,26,27], and the formed Fe(II) carbonates and Fe(II) phosphates were removed using sterile 0.22 µm filters after 3 days of precipitation in the anaerobic glove box [27]. The final concentration of Fe(II) in the NRFO medium was around 5–8 mM.

The cultures were initialed by inoculating of bacterial suspensions into 20 mL of NRFO medium and then incubated in the dark at 30 °C in the anaerobic chamber (N_2_:CO_2_:H_2_ = 90:5:5; Shel Lab Bactron IV, Portland, OR, USA). Meanwhile, the NRFO medium without the addition of bacterial suspension was set as the control. All the setups were prepared in three replicates. Before inoculation, the bacterial suspension was obtained by cultivating *Enterobacter* strains in the R2A agar for 3 days at 30 °C under anoxic conditions. The bacterial colonies on R2A plates were removed using anoxic sterile 0.9% NaCl (*w*/*v*), harvested at 8000 g for 10 min, washed three times with anoxic mineral medium and resuspended in 60 mL mineral medium. The cell numbers of bacterial suspension for four *Enterobacter* strains were quantified via 16S rRNA-based qPCR (Roche 480, Roche, Indianapolis, IN, USA). The information on primers and thermal cycling conditions for qPCR as described in Supplementary Table S1. The amplification was carried out in triplicates using the primer set of 515F-907R [28]. The reaction mixture contained 1 µL DNA as a template, 10 µL of SYBR 2 Premix EX Taq, 0.8 µL of each primer and 7.4 µL of ddH_2_O, and the reaction contained no DNA template in the negative control. Genomic DNA was extracted from cells of *Enterobacter* strains using FastDNA Spin Kit (MP Biomedical, France). DNA of *Enterobacter hormaechei* was used to clone these genes to prepare standard plasmids. Standard curves were produced using serial dilutions of the standard plasmids [24]. Only one peak was detected at the melting temperature (Tm) of 82.5 °C, which indicated the specificity of amplicons. We only accept the reactions with efficiencies ranging from 90% to 110% [24].

### 4.3. PCR

For the *Enterobacter* strains, the nitrogen cycling genes, including *napA* (periplasmic-bound nitrate reductase) [29], *narG* (nitrate reductase) [30], *nasA* (assimilatory nitrate reductase) [31], *nirK* (copper-containing nitrite reductase) [14], *nirS* (cytochrome cd1-containing nitrite reductase) [32], *norB* (nitric oxide reductase) [33] and *nosZ* (nitrous oxide reductase) [34] were determined using PCR. The information about primer sets and thermal cycles were detailed in Supplementary Table S1.

### 4.4. Chemical Analyses

Ferrous iron concentrations were analyzed using the modified ferrozine assay by Klueglein and Kappler [27], which, as a result, prevented oxidation of Fe(II) by the nitrite at acidic pH through ferrous iron with sulfamic acid but not HCl. In brief, 100 µL of culture suspension was mixed with 900 µL of 40 mM sulfamic acid for 1 h at room temperature [27]. The culture suspension was collected using syringes in the anaerobic chamber. An aliquot of 20 µL of ferrous iron extract was added with 180 µL of ferrozine solution (1 g ferrozine in 50 mM HEPES buffer, Ph = 7), which was followed by the formation of ferrous complex quantified at 562 nm using UV/Vis spectrometer (Thermo Scientific Varioskan LUX, ThermoFisher, Waltham, MA, USA). The concentrations of NO_3_^−^ and NO_2_^−^ were analyzed with ion chromatography (Dionex ICS-3000 system, Diones, Sunnyvales, CA, USA). Gas chromatography (Agilent-7890, Agilent Technologies, Santa Clara, CA, USA) was employed to determine headspace N_2_O.

### 4.5. Phenotypic Analysis

Cell morphology of *Enterobacter* spp. was characterized after growth of *Enterobacter* spp. on R2A media or NRFO medium using scanning electron microscopy (SEM, S-4800, Hitachi). Cell samples for SEM analysis were prepared in the anaerobic chamber as described before [11]. In brief, the cell samples were centrifuged for 5 min at 2000 rpm and supernatants were then discarded. After being fixed in 3% (*v*/*v*) glutaraldehyde buffered with 0.1 M sodium phosphate buffer (pH 7.2) for 1 h at room temperature, cells of *Enterobacter* strains were then washed three times in sodium phosphate buffer through centrifuged for 5 min at 2000 rpm, dehydrated in a graded alcohol series and dried using Critical Point Dryers (Leica EM CPD300, Weztlar, Germany) for 6 h. The dried cells were mounted onto a stub using double-sided carbon tape and then coated with a thin layer of gold. The samples were examined using a scanning electron microscope (Merlin compact, Zeiss, Germany).

### 4.6. Raman Spectroscopy

The cell morphology and iron(III) oxides coated on cell surfaces of *Enterobacter* strains were characterized by a confocal Raman system (Horiba Jobin Yvon S. A. S, Paris, France) set up with an integrated Olympus BXFM microscope equipped with 600 g/mm grating [35]. In brief, 2 mL of cell suspension was taken from cultures inoculated with *Enterobacter* strains after 240 h of incubation in the anaerobic chamber. After mixing an equal volume of cell suspension and anoxic D_2_O (D-99.9% atom%; Sigma-Aldrich), all the setups were anaerobically incubated for 48 h in the dark at 30 °C. Cells sampled from NRFO medium were washed with sterile ddH_2_O three times by centrifugation at 2000× *g* for 3 min. Then, cells were suspended in the ddH_2_O again, and 10 μL of them was transferred to the test slide and dried for 30 min at room temperature under anoxic conditions. The provided excitation was a 532 nm laser with the power of 50 μW on the cell samples. A 100× objective lens (Olympus) was employed to collect the Raman signal. The axial and lateral resolutions were ca. 2 μm and ca. 1 μm, respectively. The acquisition time of 1 s and a Raman spectrum ranging from 500 to 2500 cm^−1^ was used during the operation. The wavelength was calibrated by focusing the laser (532 nm) beam on a silicon wafer with a 100× objective, which showed the first-order phonon band of silicon at 520.6 cm^−1^. Generally, a total of twenty Raman spectra were collected from different areas selected randomly on each cell sample.

### 4.7. Phylogenetic Analysis

In order to investigate the potential ability of nitrate reduction in *Enterobacter* strains, nitrate reductases were checked through the NCBI database based on their genome information and published literature. A total of 28 strains were identified to possess the relevant genes such as *narI*, *narH*, *narG*, and *narZ*, and the 16S *rRNA* gene sequences of *Enterobacter* strains containing these genes were retrieved from the NCBI database. Alignment of 16S *rRNA* genes was performed using Silva (https://www.arb-silva.de/aligner/, accessed on 23 March 2022) and a phylogenetic tree was calculated with MEGA version 5.0s based on neighbor-joining methods (bootstrap values, 1000 replications) [36,37,38].

## 5. Conclusions

Typical nitrate reducers, four *Enterobacter* strains including *E. hormaechei*, *E. tabaci*, *E. mori* and *E. asburiae*, displayed a mixed biotic and chemical oxidation of iron(II) coupled to nitrate reduction. A total of 22~29% of iron(II) oxidation was roughly calculated to be linked to nitrate reduction through enzymatic NRFO by *Enterobacter* strains. Cell encrustation with iron(III) oxides and metabolic inactivity were observed for these four *Enterobacter* strains during the NRFO process after 96-h incubation. Genes encoding respiratory and periplasmic nitrate reductases were ubiquitously possessed by genomes of *Enterobacteriaceae* bacteria. They may result in the chemical reaction between nitrite and iron(II), which was one of the culprits for the cell iron(III) mineral coating and metabolic inactivity during the NRFO process. Therefore, this study indicated the interplay between microbial nitrate reduction and iron(II) oxidation by nitrate reducers, leading to the inhibition of metabolic activity for cells. Overall, this study suggests an underestimated contribution of ubiquitous nitrate reducers to biochemical iron cycling in environments.

## Figures and Tables

**Table 1:** Information of *Enterobacter* strains.

Strain	Culture Preservation Organization	Isolation Source
*E. hormaechei*	CGMCC 1.10608^T^	Pig farm
*E. tabaci*	CGMCC 1.15707^T^	Stem of a tobacco plant
*E. mori*	CGMCC 1.10322^T^	Diseased mulberry roots
*E. asburiae*	JCM 6051	Mulberry

**Table 2:** Genes linked to denitrification and nitrate reduction harbored in the genomes of strains affiliated with *Enterobacter*.

Gene	PCR Products	*E. hormaechei*	*E. tabaci*	*E. mori*	*E. asburiae*
**napA**	1040 bp	−	−	−	−
**narG**	650 bp	+	+	+	+
**nasA**	700 bp	+	+	+	+
**nirK**	526 bp	+	+	+	+
**nirS**	774 bp	+	+	+	+
**norB**	669 bp	+	+	+	+
**nosZ**	300 bp	+	+	+	+

## Supplementary Materials

The following supporting information can be downloaded at: https://www.mdpi.com/article/10.3390/molecules27175581/s1, Supplementary Table S1. Primers and qPCR processes used in this study. Supplementary Table S2. Information about *16S rRNA* of *Enterobacter* strains containing nitrate-reducing genes such as *narI*, *narH*, *narG* and *narZ*. Supplementary Table S3. Information about bacteria possessing nitrate-reducing genes based on NCBI database. Supplementary Figure S1. The culture of *Enterobacter* strains in the NRFO medium after 10-day incubation. 0# represented the abiotic setup, and 2#, 3#, 4# and 5# represented the biotic setups amended with *E. hormaechei*, *E. tabaci*, *E. mori* and *E. asburiae*, respectively. Supplementary Figure S2. Raman spectra of *Enterobacter* strains cultured in the NRFO medium after 240-h incubation. Supplementary Figure S3. The abundance of Enterobacteriaceae bacteria possessing nitrate-reducing genes based on NCBI database.

## Sample Availability

Samples of the compounds are not available from the authors.
