# Supplementary material for: Underestimation about the Contribution of Nitrate Reducers to Iron Cycling Indicated by Enterobacter Strain
Source: Molecules. 2022 Aug 30;27(17):5581. doi: 10.3390/molecules27175581 (PMC9457790; doi:10.3390/molecules27175581)
Supplement: Supplementary file 1 [file molecules-27-05581-s001.zip › Supplementary_Material2022.06.28_clean version.pdf]

Supplementary Material

Underestimation about the contribution of nitrate reducers to iron cycling indicated by *Enterobacter* strain

Ming-Jun Li[1], Meng-Yun Wei[2], Xiao-Ting Fan[2], and Guo-Wei Zhou[1*]

[1]School of Resources and Environmental Engineering, Anhui University, Hefei 230601, China

[2]Key Lab of Urban Environment and Health, Institute of Urban Environment, Chinese Academy of Sciences, Xiamen 361021, China

***Corresponding Author**

Guowei Zhou;

E-mail: gwzhou@ahu.edu.cn**;**

Phone: (+86) 551 63861441;

Fax: (+86) 551 63861441.

Supplementary Table S1. Primers and qPCR processes used in this study.

Target-genes	Function	Primer	Primers (sequences 5’-3’)	Annealing temperature	References
napA	Nitrate reductase	*NapA-V16cf*<br>*NapA-V17cr*	GCNCCNTGYMGNTTYTGYGG<br>RTGYTGRTTRANCCATNGTCCA	48ºC	(Christopher et al. 2000)
narG	Nitrate reductase	*NarG-1960f*<br>*NarG-2650r*	TAYGTSGGSCARGARAA<br>TTYTCRTACCABGTAGC	55ºC	(Laurent et al. 2002)
nasA	Nitrate reductase	*nas964*<br>*nasA1735*	CARCCNAAYGCNATGGG<br>ATNGTRTGCCAYTGRTC	58 ºC	(Allen et al. 2001)
nirK	Nitrite reductase	*NirKF*<br>*NirKR*	GGMATGGTKCCSTGGCA<br>AACTTGCCGTVGYCCAGAC	57ºC	(Paolina et al. 2010)
nirS	Nitrite reductase	*NirS-832F*<br>*NirS-1606R*	TAYCACCCSGARCCGCGCGT<br>AGKCGTTGACTTKCCGGTCG	54ºC	(Braker et al. 1998)
norB	Nitric oxidereductase	*NorBF*<br>*NorBR*	CGNGARTTYCTSGARCARCC<br>CRTADGCVCCRWAGAAVGC	55ºC	(Paolina et al. 2010)
nosZ	Nitrous oxide reductase	*NosZF*<br>*NosZR*	CCCGCTGCACACCRCCTTCGA<br>CGTCGCCSGAGATGTCGATCA	58ºC	(Throbäck et al. 2004)

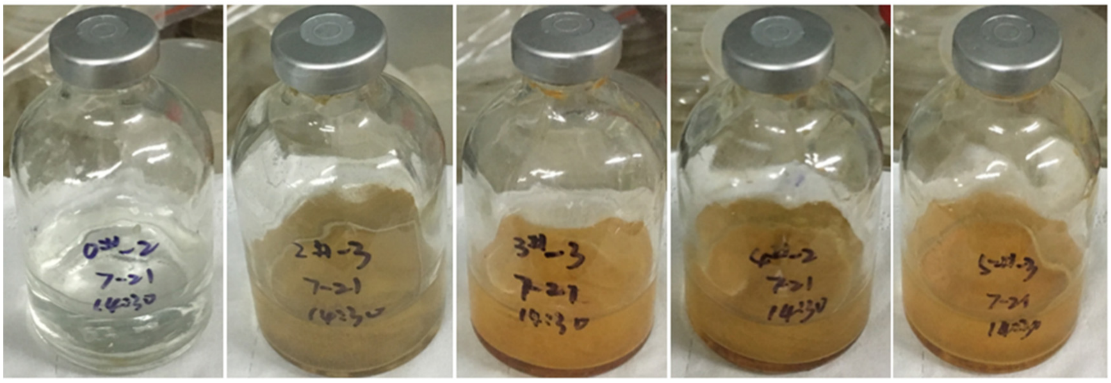

Supplementary Figure S1. The culture of *Enterobacter* strains in the NRFO medium after 10-day incubation. 0# represented the abiotic setup, and 2#, 3#, 4# and 5# represented the biotic setups amended with *E. hormaedei*, *E. tabaci*, *E. mori* and *E. asburiae*, respectively.

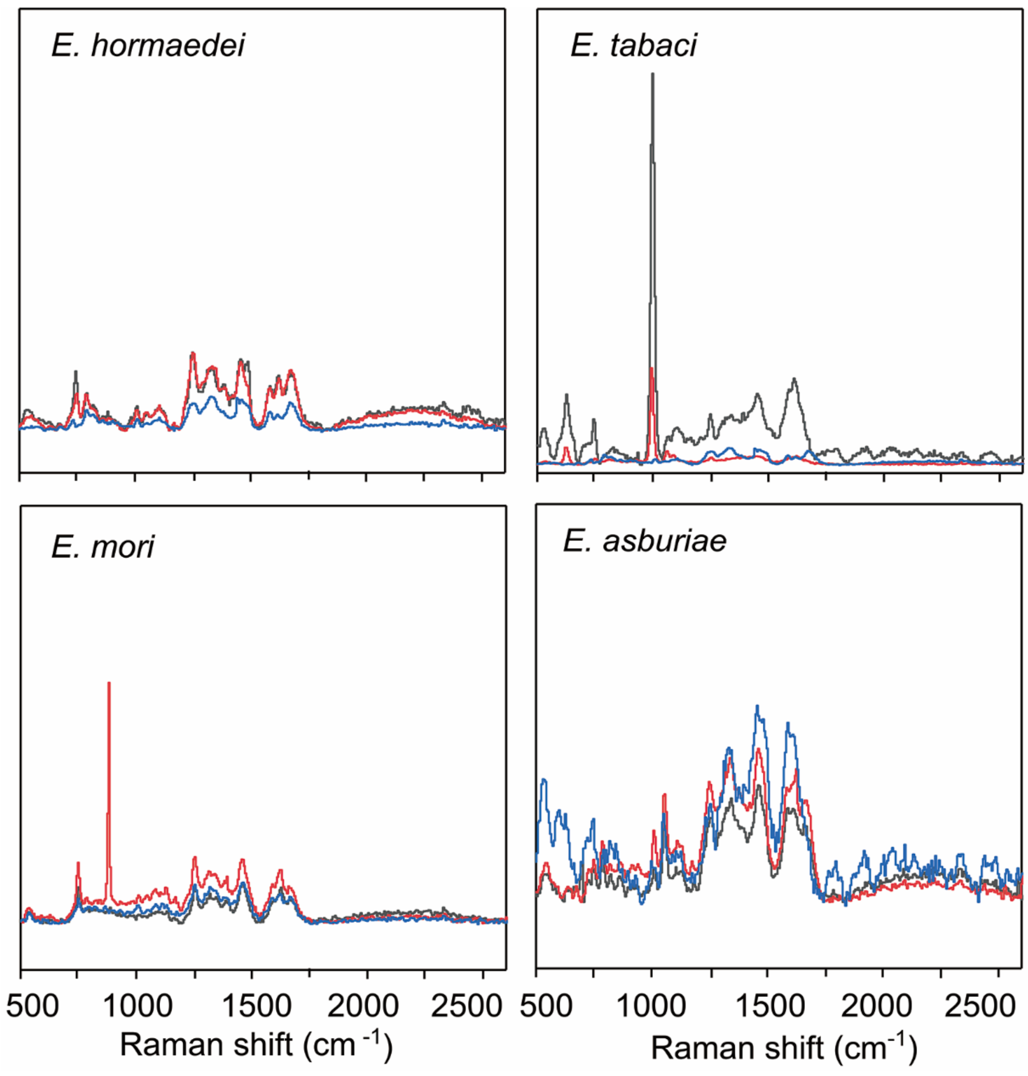

Supplementary Figure S2. Raman spectra of *Enterobacter* strains cultured in the NDFO medium after 240-h incubation. Spectra of three cells marked lines in red, blue and black for each strain were randomly chosen to be presented in the graphs.

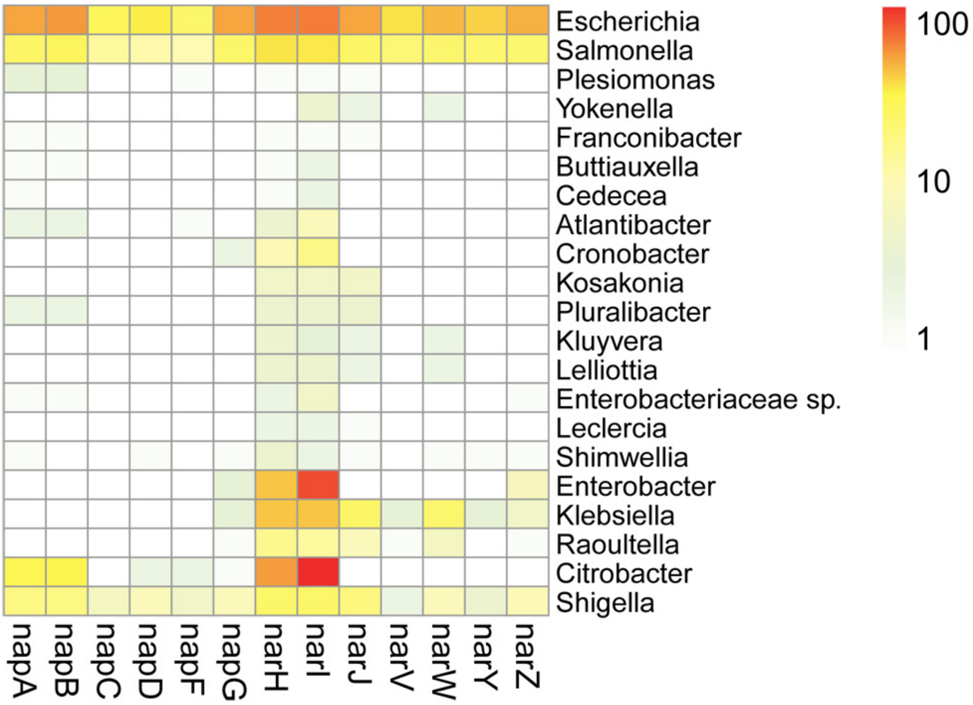

Supplementary Figure S3. The abundance of Enterobacteriaceae bacteria possessing nitrate-reducing genes based on NCBI database. The color represented the numbers of species carrying the relevant genes in the genomes of microorganisms.

References

Allen, A. E., Booth, M. G., Frischer, M. E., Verity, P. G., Zehr, J. P., and Zani, S. (2001). Diversity and detection of nitrate assimilation genes in marine bacteria. Appl. Environ. Microbiol. 67, 5343-5343.

Braker, F., Fesefeldt, A., and Witzel, K.-P. (1998). Development of PCR primer systems for amplification of nitrite reductase genes (*nirK* and *nirS*) to detect denitrifying bacteria in environmental samples. Appl. Environ. Microbiol. 64, 3769-3775.

Christopher, M. D., Paul, B., Blackall, L. L., and Mcewan, A. G. (2000). Aerobic nitrate respiration in a nitrite-oxidising bioreactor. FEMS Microbiol. Lett. 184, 113-118.

Laurent, P., Séverine, P., Fabrice, M. L., Stéphanie, H., and Jean Claude, G. (2002). Molecular analysis of the nitrate-reducing community from unplanted and maize-planted soils. Appl. Environ. Microbiol. 68, 6121-6128. doi: DOI: 10.1128/AEM.68.12.6121-6128.2002.

Paolina, G., Baggs, E. M., and Prosser, J. I. (2010). Phylogeny of nitrite reductase (nirK) and nitric oxide reductase (*norB*) genes from *Nitrosospira* species isolated from soil. FEMS Microbiol. Lett.83-89.

Throbäck, I. N., Enwall, K., Jarvis, Å., and Hallin, S. (2004). Reassessing PCR primers targeting *nirS*, *nirK* and *nosZ* genes for community surveys of denitrifying bacteria with DGGE. FEMS Microbiol. Ecol. 49, 401-417. doi: 10.1016/j.femsec.2004.04.011.
